# Effect of Neurologic Conditions on Delirium Duration and Time to ICU Discharge

**DOI:** 10.1097/CCE.0000000000001367

**Published:** 2026-01-26

**Authors:** Kate J. Frost, Heidi Chen, Zackary Schoonover, Rameela Raman, Chevis N. Shannon, Pratik P. Pandharipande, Heidi A.B. Smith

**Affiliations:** 1 Department of Anesthesiology, Perioperative and Pain Medicine, Brigham and Women’s Hospital, Boston, MA.; 2 Department of Biostatistics, Vanderbilt University, Nashville, TN.; 3 OMS-II at Edward Via College of Osteopathic Medicine, Blacksburg, VA.; 4 Department of Neurologic Sciences, Heersink School of Medicine, University of Alabama, Birmingham, AL.; 5 Department of Anesthesiology, Vanderbilt University Medical Center, Nashville, TN.; 6 Department of Pediatrics, Vanderbilt University Medical Center, Nashville, TN.

**Keywords:** acute brain dysfunction, brain injury, coma, delirium, developmental delay, ICU outcomes, neurologic intensive care unit

## Abstract

**IMPORTANCE:**

Delirium is prevalent and associated with poorer clinical outcomes in critically ill children.

**OBJECTIVES:**

We sought to determine whether presence of baseline developmental delay (DD) or a primary admission diagnosis of an acute neurologic condition (ANC) was associated with longer delirium duration and/or time to ICU discharge, and whether delirium acts as a mediator among observed outcome associations.

**DESIGN, SETTING, AND PARTICIPANTS:**

Post hoc analysis of a prospective, observational study in patients 6 months to 5 years old admitted to a tertiary PICU regardless of admission diagnosis.

**MAIN OUTCOMES AND MEASURES:**

Patients assessed daily for delirium using the Pediatric Confusion Assessment Method for the ICU series (PEDs CAM-ICU). Baseline demographics and in-hospital outcomes obtained.

**RESULTS:**

Of 282 patients, 79 had baseline DD and 54 were admitted with an ANC. Delirium prevalence among patients with DD was 53% and 43% in those with an ANC. DD was associated with significantly longer delirium duration (*p* = 0.008), with a meaningful association between ANC and delirium duration (*p* = 0.056). DD was significantly associated with a lower likelihood of ICU discharge (hazard ratio, HR, 0.76 [95% CI, 0.54–0.95]), with delirium partially mediating this relationship. Patients with ANC diagnoses trended toward a relevant association with lower likelihood of ICU discharge (HR 0.73 [0.53–1.00]) with partial delirium mediation.

**CONCLUSIONS AND RELEVANCE:**

Baseline DD among critically ill infants and children is linked to longer delirium duration and lower likelihood of ICU discharge. An innovative finding is that delirium mediates this relationship. Although ANCs were loosely correlated with both prolonged delirium duration and decreased likelihood of ICU discharge, the true impact of delirium on these patients warrants further investigation. Finally, a focus on how to mitigate the impact of DD (predisposing risk factor) on ICU delirium and outcomes in critically ill infants and children is paramount.

**KEY POINTS**

**Question:** Determine whether baseline developmental delay (DD) or an acute neurologic condition (ANC) is associated with delirium duration and/or time to ICU discharge.

**Findings:** Post hoc analysis of a prospective, observational study in 282 patients 6 months to 5 years old, assessed daily for delirium using the Pediatric Confusion Assessment Method for the ICU series, with delirium prevalence 53% among patients with DD and 43% with an ANC. DD was significantly associated with prolonged delirium duration and decreased likelihood of ICU discharge, significantly mediated by delirium. ANCs had a meaningful association with delirium duration but no association with ICU discharge.

**Meaning:** Predisposing and precipitating factors have independent associations with delirium duration and in-hospital outcomes with mediation by delirium, suggesting robust delirium screening and preventative strategies may further mitigate poorer outcomes.

## BACKGROUND

Delirium is a state of acute brain dysfunction and is common among critically ill children, with a reported prevalence between 5% and 66% with higher reported rates in patients younger than 2 years old (1–5). Delirium in critically ill pediatric patients is associated with prolonged mechanical ventilation, increased length of hospitalization, higher cost, and greater mortality (6–8). Risk factors for the development of delirium in pediatric patients include greater benzodiazepine and opioid exposure, younger age, need for mechanical ventilation, history of cyanotic heart disease, use of physical restraints, and DD (7, 9–12). In critically ill adult patients, delirium is associated with long-term cognitive impairment (13), and acute neurologic injury is an independent risk factor for delirium and longer ICU and hospital length of stay (LOS) as well as long-term cognitive impairment (14–18).

In the pediatric patient population, the relationship between neurologic conditions and delirium is not well delineated nor is the impact of delirium on long-term cognitive impairment. In addition, prior studies have demonstrated mixed results in terms of an association between neurologic conditions and ICU LOS. Ruttimann and Pollack found that head trauma and CNS infections were associated with longer ICU LOS while other CNS diseases were associated with shorter ICU LOS (19). Graham et al (20) found that patients with congenital neurodevelopmental disorders represented nearly a quarter of all PICU admissions but did not differ in terms of LOS when compared with the general PICU population.

Delirium assessment may be challenging in patients with neurologic conditions such as baseline DD or a primary diagnosis of an acute neurologic condition (ANC). When assessing patients with DD, there is concern that they may not interact with their environment in the same way as neurotypical patients, thus patients with DD may both be more at risk for under- and over-diagnosis of delirium leading to management malalignment (21). Similarly, patients admitted to the ICU with an ANC may have both perceived and real alterations from their baseline or post-injury mental status baseline and overlap between the clinical presentation of their underlying neurologic condition and the features of delirium may also lead to under- or over-diagnosis of delirium (22, 23). However, understanding delirium and possible clinical associations in the setting of DD and ANC remains extremely important in the pediatric population. We hypothesized that if the brain is already impacted by an underlying neurologic disorder, either acutely with an ANC or more chronically with DD, it may be more at risk for further acute brain dysfunction placing that population of patients at greater risk for the development of delirium, which may then lead to poorer outcomes. In this study, we aim to characterize delirium in patients with neurologic conditions to determine whether delirium in this subset of patients is similar or dissimilar to patients who are neurotypical. Furthermore, we aimed to determine whether admission to the ICU with baseline DD or an ANC are independent risk factors for longer delirium duration and/or time to ICU discharge, and whether delirium mediates any association between neurologic conditions (DD or ANC) and poorer outcomes.

## METHODS

This was a secondary analysis of a prospective observational study of pediatric patients 6 months to 5 years old, regardless of primary diagnosis, admitted to the pediatric medical and cardiac ICUs at Monroe Carell Jr Children’s Hospital at Vanderbilt between March 2013 and October 2014 to validate the PreSchool addition to the PEDs CAM-ICU series (3, 24). Exclusion criteria included: hearing/visual impairment, non-English speaking, and cognitive development less than expected for 6 months of age, planned ICU discharge/transfer, or inability to obtain consent. This study seeks to determine whether pediatric patients admitted with either an acute neurologic critical illness (admission diagnosis) or baseline DD (predisposing factor) have poorer outcomes compared with patients without an ANC or DD. The presence of any one of the following admission diagnoses included in the original validation cohort were used to define the ANC group: status epilepticus, traumatic brain injury, anoxic brain injury, central apnea, brain mass, intracranial hemorrhage, stroke, arteriovenous malformation, hydrocephalus, and/or altered mental status. Presence of baseline DD in the original validation study was obtained via parental report upon enrollment and/or documentation within the electronic medical record (EMR). Presence of DD by parental report or via the EMR defined the DD group. The type or severity of DD was not taken into consideration for this secondary analysis.

Patients underwent once daily delirium assessments with the PEDs CAM-ICU for up to 14 days of ICU stay or until transfer/discharge from the ICU, whichever came first. The institutional review board (IRB) of Vanderbilt University Medical Center determined this study with analysis of existing data and records met criteria for exemption from federal regulations regarding the protection of human subjects and did not require informed consent (IRB Number 181907, “Characterization of ICU Delirium in Pediatric Patients Admitted with Primary Neurologic Illness,” initial approval December 20, 2018). Procedures were followed in accordance with the ethical standards of the responsible committee on human experimentation (institutional or regional) and with the Helsinki Declaration of 1975.

The datasets used and/or analyzed during the current study are available from the corresponding author on reasonable request.

**Figure 1.**
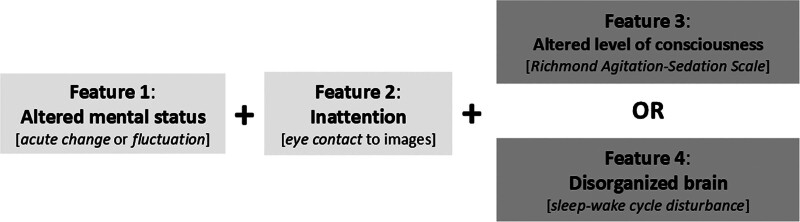
Criteria for delirium using the Pediatric Confusion Assessment Method for the ICU series (PEDs CAM-ICU). The PEDs CAM-ICU uses developmentally appropriate methods to assess the cardinal features of delirium (2, 3, 24, 27). A patient is deemed to have delirium if they exhibit an acute change or fluctuation from baseline mental status (feature 1), AND inattention (feature 2), which is assessed by the ability to maintain eye contact with mirrors and images in infants and younger children. In addition, they must EITHER have an acute altered level of consciousness in the moment (feature 3) OR disorganized brain or dysregulated systems (feature 4), defined as a sleep-wake cycle disturbance or inconsolability and unawareness of surroundings.

Upon ICU admission, demographic and risk factor data was obtained for each patient. These included age, admission diagnoses, medical history including presence of DD, as well as initial vital and laboratory values to assess severity of illness (SOI) using the Pediatric Risk of Mortality III score (25). In-hospital risk factor data and outcome data were collected daily. The accurate assessment of level of consciousness (LOC), baseline mental status and inattention is key for delirium screening, particularly in those with DD or an ANC. Participants were assessed using the Richmond Agitation-Sedation Scale (RASS) to determine LOC (26). There are four arousal categories based on the RASS: agitation (RASS +1 to +4), spontaneously alert (0), decreased arousal but responsive to voice (RASS –1 to –3), and unresponsive to voice (RASS –4 to –5). In patients who are younger or have conditions that may alter LOC (DD or ANC), sleep stage affects wakefulness; deep sleep may require stronger stimulation to distinguish from sedation. Therefore, using the PEDs CAM-ICU algorithm, if the initial RASS score was less than or equal to –3, patients received 15 seconds of physical touch, followed by 15 seconds of rest, and then reassessed using the RASS. If the second RASS was –4 or –5, they were not assessed for delirium at that time (3). If responsive to voice (RASS ≥ –3), patients were then assessed for delirium using the PEDs CAM-ICU, a bedside tool that assesses for the cardinal features of delirium as stated in the Diagnostic and Statistical Manual of Mental Disorders (DSM-5) using developmentally appropriate methods (3). For a patient to be classified as having delirium, they must have exhibited both evidence of altered mental status (feature 1), defined as an acute change or fluctuation from baseline, and inattention (feature 2), assessed by inability to maintain eye contact with images and mirrors. In addition, they must have demonstrated presence of either an acutely altered LOC (feature 3), defined as any RASS score other than zero, or the presence of dysregulated systems (feature 4), defined as a sleep-wake cycle disturbance or inconsolability and unawareness of surroundings (**Fig. 1**).

Delirium screening in patients with DD and ANC is optimized using the PEDs CAM-ICU algorithm because it is anchored on DSM-5 criteria, with a focus on cardinal features such as inattention, using objective and in the moment assessments. Differentiating the expected mental status baseline from an alteration uses communication and evaluation by the bedside nurse, medical team and/or family as outlined in feature 1 of the PEDs CAM-ICU. The PEDs CAM-ICU tool accurately separates the assessment of inattention (feature 2) from cognition allowing valid screening despite the presence of DD or other conditions, thereby decreasing bias that a positive screen for inattention is due to acute or chronic cognitive dysfunction. Validation studies of the PEDs CAM-ICU occurred in three separate cohort studies including ages 5–18 years old, 6 months to 5 years, and birth to 6 months against a reference standard (Child and Adolescent Psychiatrist), including a representative sample of all patients admitted to the ICU regardless of DD or admission diagnosis (2, 3, 27). Patients assessed to have delirium using the PEDs CAM-ICU were further classified by motoric subtype, with hypoactive delirium defined as RASS 0 to –3, and hyperactive as RASS +1 to +4.

### Statistical Analysis

**Figure 2.**
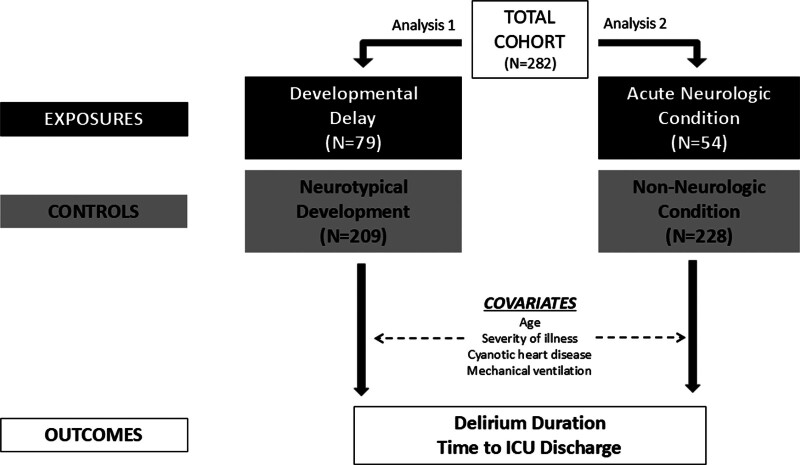
Cohort analysis overview. We performed two independent analyses dividing the cohort initially based on presence of developmental delay (DD) vs neurotypical patients and then separately based upon admission with an acute neurologic condition (ANC) vs non-neurologic conditions. We then compared these two groups to study the effects of DD or an ANC on delirium duration and/or time to ICU discharge. Key covariates were accounted for including age, severity of illness, cyanotic heart disease, and need for mechanical ventilation.

Patient characteristics, including demographics, baseline, in-hospital and outcome variable were summarized using frequencies (%) for categorical variables and medians and interquartile ranges (IQRs) or mean and sdfor continuous variables. This secondary analysis of a prospective cohort of patients younger than 5 years old admitted to the ICU was designed as two separate analyses based on independent variables (DD or ANC). Analysis 1 assessed the impact of baseline DD vs. patients with neurotypical development. Analysis 2 assessed the impact of a primary ICU admission diagnosis consisting of an ANC vs. those admitted with a non-neurologic primary diagnosis (**Fig. 2**). Patients with both DD and ANC were represented in both analyses 1 and 2.

Negative binomial regression models were used to determine the effect of baseline DD or ANC on delirium duration, defined as the number of positive delirium days during a patient’s ICU admission. Multivariable Cox proportional hazard regression models were used to determine the effect of baseline DD or ANC on time to ICU and hospital discharge. Hazard ratios (HRs) represent the likelihood of ICU discharge. Therefore, a HR greater than 1 represents a greater likelihood of ICU discharge (e.g., shorter duration of stay), whereas a HR less than 1 represents a lower likelihood of ICU discharge (e.g., longer duration of stay).

**Figure 3.**
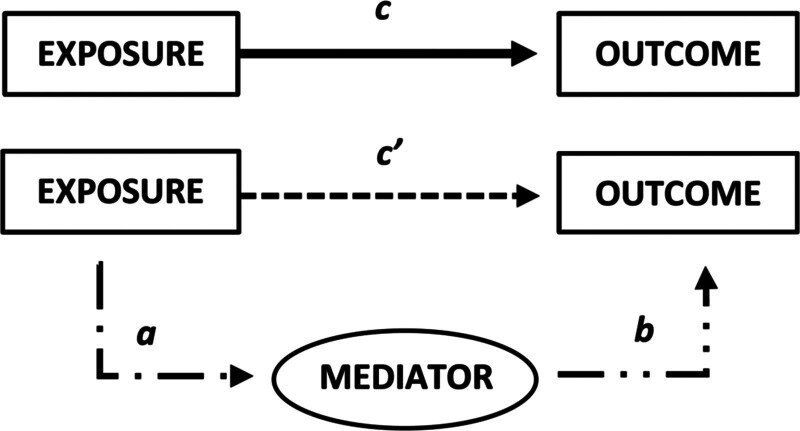
Concept of mediation. Mediation occurs when the exposure of interest is associated with the proposed mediator and that mediator is also independently associated with the outcome of interest. In this conceptual figure, *c*′ is the direct effect of the exposure on the outcome, whereas *a* is the exposure-mediator effect and *b* is the mediator-outcome effect. The total effect (*c*) *= ab + c*′, direct effect (*c*′) *= c–ab*, and indirect effect (*ab*) *= c–c*′ (27).

Mediation can occur when the exposure of interest is associated with the proposed mediator and that mediator is also independently associated with the outcome of interest. The total effect then includes both the direct effect of the exposure (e.g., DD or ANC) on the outcome of interest (e.g., time to discharge) and the indirect effect of the exposure on the outcome through the mediator (e.g., delirium) (**Fig. 3**). Mediation of possible associations between baseline DD or ANC on time to ICU discharge by delirium can be inferred by examining the statistical models with and without controlling for delirium as a covariate. When the total effect is statistically stronger than the direct effect alone, mediation (indirect effect) can be implied. All models were adjusted for possible confounders including age, SOI, history of cyanotic heart disease, and need for mechanical ventilation (28).

Analyses were performed using R-statistical software, Version 4.1.1 (www.r-project.org).

## RESULTS

### Patient Characteristics

A total of 282 patients were included in the post hoc cohort analyses. Demographic, baseline, in-hospital, and outcome variables are summarized in **Table 1**. The main analysis was undertaken using two separate analyses with the cohort divided in two ways: analysis 1–presence vs. absence of baseline DD (*n* = 79 patients), and analysis 2–presence vs. absence of a primary admission diagnosis for an ANC (*n* = 54 patients). Seven patients had both baseline DD and a primary admission diagnosis of an ANC; these patients are represented in both analyses. In analysis 1, patients with baseline DD had a higher rate of cyanotic heart disease (*p* < 0.01), need for mechanical ventilation (*p* < 0.01), and lower likelihood of hospital discharge (HR 0.72 [0.50–0.90]), compared with those with neurotypical development. However, there was no significant difference in their SOI, exposure to benzodiazepine or coma prevalence. In analysis 2, patients with a primary admission diagnosis of an ANC (*n* = 54 patients) demonstrated significantly fewer patients with cyanotic heart disease or need for mechanical ventilation but no difference in SOI, benzodiazepine exposure, or likelihood of hospital discharge (HR 0.83 [0.61–1.17]) compared with those with a non-neurologic admission diagnosis. Interestingly, patients with an ANC diagnosis did not demonstrate a higher rate of coma compared with non-neurologic diagnoses.

**TABLE 1.** Demographics, Baseline and In-hospital Variables and Outcomes

	All Patients, *n* = 282	Analysis 1		Analysis 2	
		Developmental Delay, *n* = 79	Neurotypical Development, *n* = 203	Neurologic Diagnosis, *n* = 54	Non-Neurologic Diagnosis, *n* = 228
Demographics					
Age (mo)	19.0 (10.0, 34.0)	17.0 (11.0, 30.5)	21.0 (10.0, 36.0)	27.0 (9.0, 36.0)	19.0 (10.8, 33.2)
Male	177 (63)	50 (63)	127 (63)	34 (63)	143 (63)
Baseline and in-hospital variables					
Cyanotic heart disease	88 (31)	34 (43)	54 (27)	2 (4)	86 (38)
Pediatric risk of mortality score	4.0 (0, 10.0)	4.0 (0, 10.0)	4.0 (0, 8.5)	2.0 (0, 7.8)	4.5 (0, 10.0)
Mechanically ventilated	143 (51)	50 (63)^a^	93 (46)^a^	20 (37)^a^	123 (54)^a^
Benzodiazepine exposure (mg)	0.0 (0.0, 3.5)	0.0 (0.0, 2.4)	0.3 (0.0, 8.0)	0.0 (0.0, 0.9)	0.0 (0.0, 5.1)
Ever comatose	79 (28)	28 (35)	51 (25)	12 (22)	67 (29)
Outcome variables					
ICU length of stay (d)	4.0 (3.0, 9.8)	7 (3.5, 13.5)^a^	4.0 (2.0, 7.5)^a^	4.5 (2.3, 9.0)	4.0 (3.0, 10.0)
Mechanical ventilation (d)^b^	4.0 (1.0, 8.0)	6.0 (1.0, 12.0)	3.0 (1.0, 8.0)	3.0 (2.0, 6.0)	4.0 (1.0, 11.5)
Hospital length of stay (d)	7 (3, 16)	12 (6, 26)^a^	5 (3, 13)^a^	7 (3, 16)	7 (3, 16)
Delirium duration (d)^b^	1 (1, 2)	2 (1, 3)^a^	1 (1, 2)^a^	1 (1, 2)	1 (1, 2)
Delirium prevalence	127 (45)	42 (53)	85 (42)	23 (43)	104 (46)
In-hospital mortality	7 (2)	6 (8)^a^	1 (0)^a^	0 (0)	7 (3)

Data are presented as number of patients (%) or medians with interquartile range.

a Indicates a statistically significant result (*p* < 0.05) between two groups within each analyses 1 and 2.

b Outcome based on only those exposed (delirium present or need for mechanical ventilation).

### Outcomes

The prevalence of delirium in the combined cohort was 45%, with prevalence rates of 53% in patients with DD and 43% in those admitted with ANC. The most common delirium subtype was hypoactive (74%) with similar rates in those with DD (72%) and ANC (70%). In the total cohort, the median (IQR) delirium duration was 1 day (1–2 d). Baseline DD was significantly associated with longer delirium duration (Relative Risk [RR] 1.62, 95% CI [1.13–2.32]), whereas an ANC diagnosis did not have a statistically significant association with longer delirium duration ANC (RR 1.57, 95% CI [0.98–2.48]).

**Figure 4.**
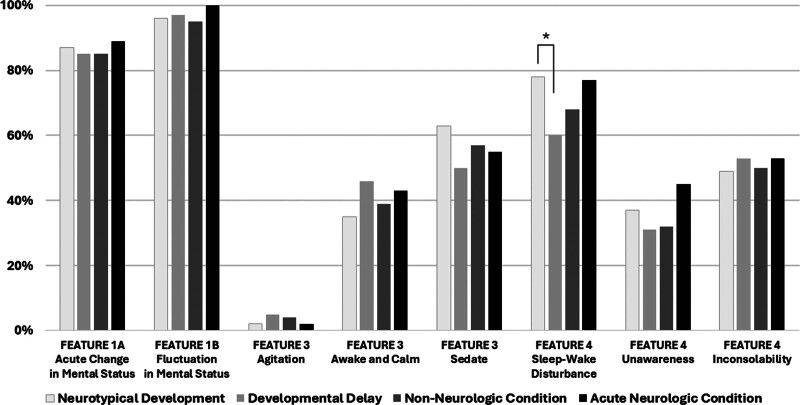
Delirium characteristics in pediatric patients. The four features of delirium as assessed by the Pediatric Confusion Assessment Method for the ICU series (PEDs CAM-ICU) demonstrated similar characteristics among patients with baseline developmental delay (DD) vs neurotypical development, as well as in patients admitted with an acute neurologic condition vs non-neurologic conditions. Patients with baseline DD had significantly lower occurrence of sleep-wake cycle disturbances compared with neurotypical patients. *p < 0.01.

The four features of delirium as assessed by the PEDs CAM-ICU series include acute change or fluctuation from baseline mental status, inattention, altered consciousness in the moment, and system dysregulation (sleep-wake cycle disturbance, inconsolability, unawareness of surroundings) (2, 3, 24, 27). We explored the patterns of delirium features present in patients with and without baseline DD and in patients with and without an ANC ICU admission diagnosis (**Fig. 4**). In analysis 1, patients with DD had significantly lower rates of sleep-wake cycle disturbances (*p* < 0.01). Interestingly, among patients with inattention (less eye contact to mirrors/pictures), neurotypical patients demonstrated significantly lower scores (median 1.5, IQR 0.0–5.0) compared with patients with DD (median 3.5, IQR 0.0–6.0) (*p* < 0.01). In analysis 2, patients with an ANC admission diagnosis had similar patterns of delirium characteristics when compared with those with non-neurologic admission diagnoses.

**Figure 5.**
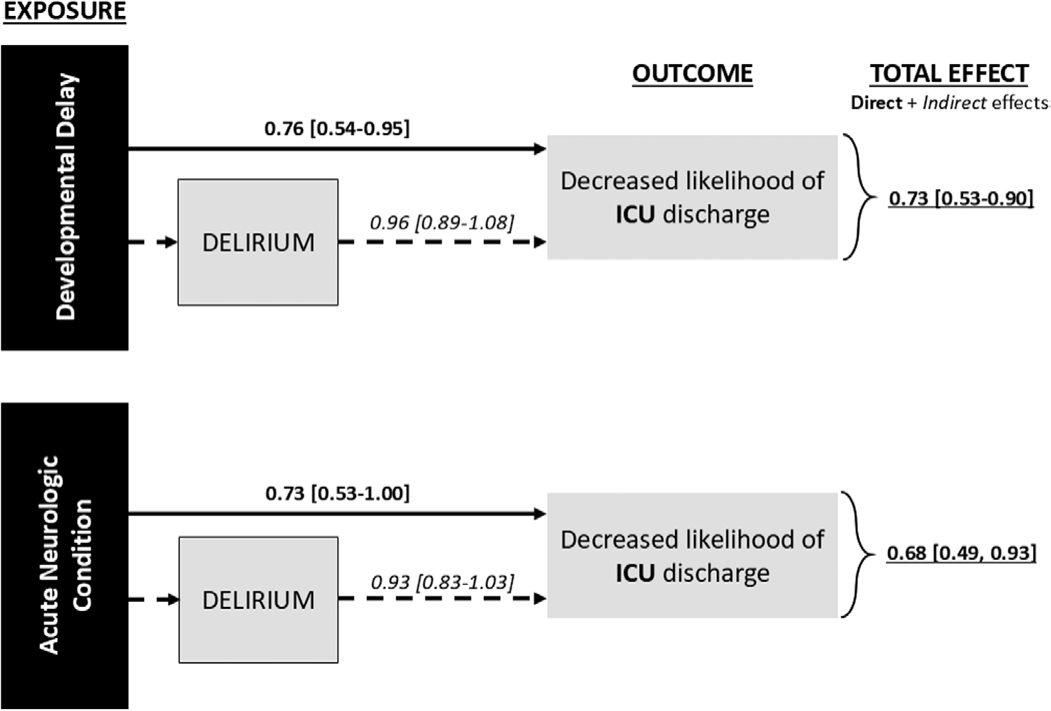
Outcomes impacted by delirium and neurologic conditions. Developmental delay (DD) is associated with a decreased likelihood of ICU discharge (longer ICU stay). Acute neurologic condition (ANC) diagnosis has a less meaningful association with a decreased likelihood of ICU discharge. To determine mediation, delirium was removed as a covariate from the model, and the total effect was calculated. After this, the relationships strengthened, as indicated by a decreased hazard ratio (HR) and narrowed CI. Therefore, the total effect between DD and ANC and time to ICU discharge is not explained by the direct effect alone, and, thus, the indirect effect, or mediation by delirium, can be inferred. Reported are HR (95% CI). HR represents the likelihood of ICU, where a HR greater than 1 represents a greater likelihood of discharge, whereas a HR less than 1 represents a lower likelihood of discharge.

DD was significantly associated with a decreased likelihood of ICU discharge (HR 0.76 [95% CI, 0.54–0.95]) with delirium mediating this relationship (**Fig. 5**). ANC diagnosis trended toward a meaningful association with lower likelihood of ICU discharge (HR 0.73 [95% CI, 0.53–1.0]) with partial delirium mediation but was not statistically significant. In-hospital mortality was 2% (7 deaths) within the full cohort with a significantly higher proportion (8%, 6 deaths) within the subgroup with baseline DD (*p* < 0.001).

## DISCUSSION

Neurologic conditions, whether acute or chronic in nature, may increase susceptibility of the brain to delirium and worse outcomes during critical illness. The presence of DD is a common factor that pediatric clinicians must consider when caring for infants and children. We demonstrate that DD is significantly associated with longer duration of delirium and lower likelihood of ICU discharge (longer ICU stay). Furthermore, we highlight the role delirium plays through mediation to negatively impact or exacerbate worse outcomes as seen between DD and time to ICU discharge. These results emphasize the importance of routine monitoring of delirium and implementation of preventative management strategies for delirium (using an appropriate amount of sedation, careful selection of sedating medications, attempting to maintain a normal sleep/wake cycle, etc.) to mitigate risks of poorer outcomes particularly in pediatric patients with neurologic conditions.

There remain questions regarding accuracy of delirium monitoring among patients with DD. This study demonstrates similarities in the patterns of delirium characteristics among neurotypical patients and those with DD. In the assessment of inattention, a cardinal feature of delirium, the PEDs CAM-ICU requires eye contact to pictures/mirrors as an objective measure of sustained attention in patients developmentally younger than 5 years old. Among patients with inattention (eye contact to < 8 pictures/mirrors), neurotypical patients were more likely to attend to only 1–2 cards whereas patients with DD were more likely to attend to 3–4 cards despite having no significant difference in LOC during the assessment. The overriding concern is whether patients with DD can consistently interact and respond to assessments reflective of delirium rather than cognitive impairment. However, when a delirium tool accurately separates the assessment of cognition from inattention as the PEDs CAM-ICU has done, then delirium assessment can be valid and reliable despite the presence of DD or other conditions that may be associated with decreased cognition. This has similarly been done in patients with severe dementia using a tool that was built off the PEDs CAM-ICU called the 4-DSD (29). One divergent pattern of delirium characteristics was the significant increase in sleep-wake cycle disturbances exhibited among neurotypical patients compared to patients with developmental delay. This observation may be key in understanding the mechanics of sleep hygiene in this patient population and on delirium prevention.

In analysis 2, we analyzed the impact of ANCs such as status epilepticus, traumatic brain injury, or intracranial hemorrhage on delirium prevalence and key outcomes. There was no significant difference in delirium prevalence or motoric subtypes between patients admitted for an ANC vs non-neurologic condition, though a meaningful relationship was reported between ANCs and prolonged delirium duration. Admission with an ANC was not significantly associated with time to ICU discharge. Patients who are admitted with ANC are often assumed to have depressed levels of consciousness (LOC). As a continuation of this concern, the assumption is that the presence of an altered mental status limits the perceived accuracy of delirium screening in this cohort. Similar to DD, these concerns regarding the ability of patients with ANC to be assessed for delirium have delayed a full understanding of the epidemiology of delirium in these patients. This study demonstrated that patients admitted with ANC did not suffer from greater prevalence or duration of coma. Further, patients with ANC with delirium had similar patterns of features such as severity of inattention, LOC, sleep-wake cycle disturbance, inconsolability and unawareness of surroundings compared with patients with non-neurologic conditions.

Moving forward, the understanding of delirium characteristics among cohorts with acute or chronic neurologic conditions should be described in addition to reporting delirium prevalence. Another key for delirium assessment is the determination of an acute change or fluctuation from baseline mental status based on DSM-5 criteria. For patients with DD or ANC, identifying the baseline mental status may not be clear without information from a parent or primary caregiver (23). Patients that have suffered from an ANC may develop a new mental status baseline during their hospitalization due to a significant injury with an unknown recovery period, whereas other patients with a less severe ANC may return to their pre-admission baseline more quickly. Differentiating the expected mental status baseline from an alteration requires communication and collaboration with the medical team. Thus, even with information from the parent or primary caregiver, baseline mental status may be difficult to determine if the patient has developed a new “normal” baseline secondary to their injury. Despite these important considerations, success of delirium assessment with tools such as the PEDs CAM-ICU compared with reference standard psychiatry assessments is well documented (3). Routine screening paired with communication of the patient’s mental status by the rotating bedside staff and family involvement can help identify an acute fluctuation or change in mental status. Although the staff may not have a clear recovery trajectory of a patient with an ANC, in collaboration on rounds with the nurse, the care team should be able to anticipate mental status patterns, and fluctuation from a new “normal” day to day while in the hospital.

A limitation of this study is that it is a secondary analysis of a previous prospective validation study which limited the accumulation of larger subgroups that would have allowed for more robust analysis of possible associations and less likelihood of underpowered results. Furthermore, the impact of an acute or chronic neurologic conditions on the development of delirium may also differ by the severity of DD or ANC and therefore may have different risk profiles. The impact of varying degrees of DD or type of ANC was not investigated. In addition, there were seven patients that had both DD and an ANC and were represented in both analyses 1 and 2. Therefore there may be some confounding effects of DD or ANC on the other analysis. Another limitation is the presence of possible confounders that are not known and therefore could impact the strength of associations. Delirium assessments were conducted using the PEDs CAM-ICU, a validated tool using developmentally appropriate assessments based on DSM criteria. At the time of this study, the PEDs CAM-ICU was validated for use in patients who are developmentally 6 months of age and older and has subsequently been validated for use in patients from birth (neonates) to adolescence. Therefore, the understanding of delirium epidemiology and impact on outcomes was not assessed in patients younger than 6 months of age and study results cannot be fully generalized to all patients in the ICU (2, 3, 27, 30). Future studies will be able to include neonates to adolescence with objective routine delirium screening and further delineate the characteristics of delirium among all ages, those with DD, and admitted for a wide range of neurologic conditions.

## CONCLUSIONS

Baseline developmental delay among critically ill infants and children is linked to longer delirium duration. Furthermore, DD is associated with a reduced likelihood of ICU discharge, and this association is mediated by delirium, representing a novel finding. Although acute neurologic conditions were loosely correlated with both prolonged delirium duration and decreased likelihood of ICU discharge, the true impact of delirium on these patients warrants further investigation. Finally, a focus on how to mitigate the impact of DD (predisposing risk factor) on ICU delirium and outcomes in critically ill infants and children is paramount. Delirium may not only be associated with worse outcomes but also mediate the severity of those relationships and therefore earlier mitigation may have a greater impact on patient outcomes.

## ACKNOWLEDGMENTS

Center for Critical Illness, Brain Dysfunction and Survivorship (CIBS) provides research infrastructure for study conduct and mentorship, at Vanderbilt University Medical Center. Surgical Outcomes Center for Kids supports trainee research experiences at Vanderbilt University Medical Center.

## References

[1] Association AP: Diagnostic and Statistical Manual of Mental Disorders. 5th ed. Arlington, VA. American Psychiatric Publishing, 2013

[2] Smith HAB Boyd J Fuchs DC: Diagnosing delirium in critically ill children: Validity and reliability of the pediatric confusion assessment method for the intensive care unit. Crit Care Med 2011; 39:150–157 20959783 10.1097/CCM.0b013e3181feb489 PMC3776416

[3] Smith HA Gangopadhyay M Goben CM: The preschool confusion assessment method for the ICU: Valid and reliable delirium monitoring for critically ill infants and children. Crit Care Med 2016; 44:592–600 26565631 10.1097/CCM.0000000000001428 PMC4764386

[4] Silver G Traube C Kearney J: Detecting pediatric delirium: Development of a rapid observational assessment tool. Intensive Care Med 2012; 38:1025–1031 22407142 10.1007/s00134-012-2518-z

[5] Smith HAB Besunder JB Betters KA: 2022 Society of Critical Care Medicine Clinical Practice Guidelines on prevention and management of pain, agitation, neuromuscular blockade, and delirium in critically ill pediatric patients with consideration of the ICU environment and early mobility. Pediatr Crit Care Med. 2022; 23:e74–e110 35119438 10.1097/PCC.0000000000002873

[6] Smith HAB Brink E Fuchs DC: Pediatric delirium: Monitoring and management in the pediatric intensive care unit. Pediatr Clin North Am. 2013; 60:741–760 23639666 10.1016/j.pcl.2013.02.010

[7] Traube C Silver G Gerber LM: Delirium and mortality in critically ill children: Epidemiology and outcomes of pediatric delirium. Crit Care Med 2017; 45:891–898 28288026 10.1097/CCM.0000000000002324 PMC5392157

[8] Traube C Mauer E Gerber L: Cost associated with pediatric delirium in the ICU. Crit Care Med. 2016; 44:1175–1179 10.1097/CCM.0000000000002004 PMC5592112 27518377

[9] Patel AK Biagas KV Clarke EC: Delirium in children after cardiac bypass surgery. Pediatr Crit Care Med. 2017; 18:165–171 27977539 10.1097/PCC.0000000000001032 PMC5658045

[10] Smith HAB Gangopadhyay M Goben CM: Delirium and benzodiazepines associated with prolonged ICU stay in critically ill infants and young children. Crit Care Med 2017; 45:1427–1435 28594681 10.1097/CCM.0000000000002515

[11] Traube C Silver G Reeder RW: Delirium in critically ill children: An international point prevalence study. Crit Care Med 2017; 45:584–590 28079605 10.1097/CCM.0000000000002250 PMC5350030

[12] Ista E Traube C de Neef M; Dutch Multidisciplinary Pediatric Delirium Guideline Group: Factors associated with delirium in children: A systematic review and meta-analysis. Pediatr Crit Care Med 2023; 24:372–381 36790201 10.1097/PCC.0000000000003196 PMC10164044

[13] Pandharipande PP Girard TD Jackson JC; BRAIN-ICU Study Investigators: Long-term cognitive impairment after critical illness. N Engl J Med 2013; 369:1306–1316 24088092 10.1056/NEJMoa1301372 PMC3922401

[14] Patel MB Bednarik J Lee P: Delirium monitoring in neurocritically ill patients: A systematic review. Crit Care Med 2018; 46:1832–1841 30142098 10.1097/CCM.0000000000003349 PMC6185789

[15] Mitasova A Kostalova M Bednarik J: Poststroke delirium incidence and outcomes: Validation of the confusion assessment method for the intensive care unit (CAM-ICU). Crit Care Med 2012; 40:484–490 22001583 10.1097/CCM.0b013e318232da12

[16] Naidech AM Beaumont JL Rosenberg NF: Intracerebral hemorrhage and delirium symptoms. Length of stay, function, and quality of life in a 114-patient cohort. Am J Respir Crit Care Med 2013; 188:1331–1337 24102675 10.1164/rccm.201307-1256OC PMC3919076

[17] Oldenbeuving AW de Kort PL Jansen BP: Delirium in the acute phase after stroke: Incidence, risk factors, and outcome. Neurology 2011; 76:993–999 21307355 10.1212/WNL.0b013e318210411f

[18] Rosenthal LJ Francis BA Beaumont JL: Agitation, delirium, and cognitive outcomes in intracerebral hemorrhage. Psychosomatics 2017; 58:19–27 27665997 10.1016/j.psym.2016.07.004 PMC5836544

[19] Ruttimann UE Pollack MM: Variability in duration of stay in pediatric intensive care units: A multiinstitutional study. J Pediatr 1996; 128:35–44 8551419 10.1016/s0022-3476(96)70425-0

[20] Graham RJ Dumas HM O’Brien JE: Congenital neurodevelopmental diagnoses and an intensive care unit: Defining a population. Pediatr Crit Care Med 2004; 5:321–328 15215000 10.1097/01.pcc.0000128892.38431.2b

[21] Kolmar AR Paton AM Kramer MA: Differences in delirium evaluation and pharmacologic management in children with developmental delay: A retrospective case-control study. J Intensive Care Med 2024; 39:170–175 37563949 10.1177/08850666231194534 PMC10938448

[22] Ubeda Tikkanen A Kudchadkar SR Goldberg SW: Acquired brain injury in the pediatric intensive care unit: Special considerations for delirium protocols. J Pediatr Intensive Care 2021; 10:243–247 34745696 10.1055/s-0040-1719045 PMC8561795

[23] Bieber ED Smith HAB Fuchs DC: Altered mental status and delirium in pediatric patients. Semin Neurol 2024; 44:707–719 39348852 10.1055/s-0044-1791227

[24] Madden M Smith H Williams S: Delirium. In: Current Concepts in Pediatric Critical Care. Adeyinka A Irving S Rehder K, (eds). Mount Prospect, Ill. Society of Critical Care Medicine, 2025, pp 171–186

[25] Pollack MM Patel KM Ruttimann UE: PRISM III: An updated pediatric risk of mortality score. Crit Care Med 1996; 24:743–752 8706448 10.1097/00003246-199605000-00004

[26] Sessler CN Gosnell MS Grap MJ: The Richmond Agitation-Sedation Scale: Validity and reliability in adult intensive care unit patients. Am J Respir Crit Care Med 2002; 166:1338–1344 12421743 10.1164/rccm.2107138

[27] Canter MO Tanguturi YC Ellen Wilson J: Prospective validation of the preschool confusion assessment method for the ICU to screen for delirium in infants less than 6 months old. Crit Care Med 2021; 49:e902–e909 34166285 10.1097/CCM.0000000000005099

[28] Rijnhart JJM Lamp SJ Valente MJ: Mediation analysis methods used in observational research: A scoping review and recommendations. BMC Med Res Methodol 2021; 21:226 34689754 10.1186/s12874-021-01426-3 PMC8543973

[29] Morandi A Grossi E Lucchi E: The 4-DSD: A new tool to assess delirium superimposed on moderate to severe dementia. J Am Med Dir Assoc 2021; 22:1535–1542.e3 33823162 10.1016/j.jamda.2021.02.029

[30] Ely EW Margolin R Francis J: Evaluation of delirium in critically ill patients: Validation of the confusion assessment method for the intensive care unit (CAM-ICU). Crit Care Med 2001; 29:1370–1379 11445689 10.1097/00003246-200107000-00012

